# Retinal vessel geometry in patients with idiopathic epiretinal membrane

**DOI:** 10.1038/s41598-023-32025-5

**Published:** 2023-03-29

**Authors:** Eun Kyoung Lee, Hye Jee Kim, Sang-Yoon Lee, Su Jeong Song, Hyeong Gon Yu

**Affiliations:** 1 Department of Ophthalmology, Seoul National University College of Medicine, Seoul National University Hospital, #101, Daehak-Ro, Jongno-Gu, Seoul, 03080 Republic of Korea; 2 Seoul Shinsegae Eye Clinic, Seoul, Korea; 3 Department of Ophthalmology, Kangbuk Samsung Hospital, Sungkyunkwan University School of Medicine, Seoul, Korea; 4 Retina Center, Sky Eye Institute, Seoul, Korea

**Keywords:** Medical research, Pathogenesis

## Abstract

We investigated the associations between retinal vascular geometric measurements and idiopathic epiretinal membrane (ERM). Whether changes in retinal vascular geometry are independent of systemic cardiovascular risk factors was also evaluated. This retrospective, cross sectional study included 98 patients with idiopathic ERM, and 99 healthy age-matched controls. Quantitative retinal vascular parameters were measured from digital retinal fundus photographs using a semi-automated computer-assisted program. Multivariate logistic regression analyses were performed to evaluate associations between retinal vascular geometric parameters and the presence of idiopathic ERM after adjusting for systemic cardiovascular risk factors. There was no significant difference in the baseline characteristics of the two groups, except that the ERM group had a higher proportion of females than the control group. In multivariate regression analyses, female sex (odds ratio [OR] 0.402; 95% CI 0.196–0.802; *P* = 0.011), wider retinal venular caliber (OR 16.852; 95% CI 5.384–58.997; *P* < 0.001) and decreased total fractal dimension (OR 0.156; 95% CI 0.052–0.440; *P* = 0.001) were associated with idiopathic ERM. Idiopathic ERM was associated with alterations in global retinal microvascular geometric parameters, wider retinal venules, and less complex vascular branching patterns, independent of cardiovascular risk factors.

## Introduction

Idiopathic epiretinal membrane (ERM) is defined as a fibrocellular proliferation on the inner retinal surface of the macular area without any associated ocular abnormalities^1^. ERM may cause visual impairments and metamorphopsia, and may cause retinal vessels to stretch or contract along with neural tissue. Movement or shift of retinal vessels in patients with ERM has been described in several reports^2,3^. Morphological changes in the foveal capillary architecture that occur due to traction caused by ERM have been studied recently using optical coherence tomography angiography^4^. Furthermore, epidemiologic studies of the prevalence of ERM have described hypercholesterolemia^5,6^ and narrower retinal arteriolar diameter^7^ as risk factors for ERM.

A thorough evaluation of the retinal vascular geometry may provide clues to the association between retinal microvascular abnormalities and ERM. Newer retinal parameters such as fractal dimension, tortuosity, bifurcation/branching angle, and retinal vascular caliber using Singapore I Vessel Assessment (SIVA, cloud-based version, National University of Singapore, Singapore), a semi-automated software are now available to assess the retinal vascular geometry, and are a reflection of the efficiency and optimal functioning of microcirculation in the retinal network^8^. Previous studies have shown that these geometric retinal vascular parameters are associated with hypertension^9^, and Alzheimer’s disease^10^. Nevertheless, the association between retinal vascular parameters using SIVA software and idiopathic ERM has not been investigated.

In this study, we quantitatively measured retinal vascular geometric parameters in eyes with idiopathic ERM and analyzed which retinal vascular geometric parameters are associated with idiopathic ERM. We also sought to determine whether these changes in retinal vessel geometry was independent of systemic cardiovascular risk factors.

## Results

Ninety-eight eyes were included in the idiopathic ERM group and 99 eyes were included in the control group. Table 1 outlines the summary of the patients’ demographics and baseline clinical characteristics. There were no statistically significant differences in the prevalence of hypertension, diabetes, and dyslipidemia, or in body mass index (BMI) values of the two groups. The idiopathic ERM group had a higher proportion of female patients than the control group (*P* = 0.015).

**Table 1:** Baseline characteristics of patients with epiretinal membrane and normal controls.

Variable	Idiopathic ERM (98 eyes)	Controls (99 eyes)	*P* value
Age (yrs)	63.3 ± 7.5	64.4 ± 10.6	0.397*
Sex (male:female)	29:69	47:52	**0.015**^**†**^
RE:LE	42:56	50:49	0.351^†^
HTN, n (%)	32 (32.7%)	26 (26.3%)	0.408^†^
DM, n (%)	14 (14.3%)	20 (20.2%)	0.363^†^
Hypertriglyceridemia	10 (10.2%)	12 (12.1%)	0.841^†^
Hypercholesterolemia	27 (27.6%)	18 (18.2%)	0.163^†^
BMI	24.18 ± 2.95	24.21 ± 2.94	0.950*

*ERM* epiretinal membrane, *yrs* years, *RE* right eye, *LE* left eye, *HTN* hypertension, *DM* diabetes mellitus, *BMI* body mass index.

Continuous variables are reported as mean ± standard deviation (range). All other data are numbers.

Significant factors appear in boldface.

*Student *t* test.

^†^Chi-square test.

Table 2 shows the comparison of retinal parameters between the idiopathic ERM and control groups. Compared with the controls, the idiopathic ERM group had wider arteriolar (163.56 ± 14.15 vs. 158.61 ± 10.42 µm, *P* = 0.006) and venular calibers (211.64 ± 15.87 vs. 199.79 ± 15.71 µm, *P* < 0.001). The idiopathic ERM group had smaller total (1.333 ± 0.059 vs. 1.355 ± 0.075, *P* = 0.022) and arteriolar fractal dimensions (1.146 ± 0.072 vs. 1.181 ± 0.092, *P* = 0.003) than the control group. Venular fractal dimensions were not different between the groups (*P* = 0.638). Furthermore, the idiopathic ERM group had more tortuous venules (0.593 ± 0.137 vs. 0.544 ± 0.119 [× 10^–4^], *P* = 0.008) and larger venular branching angles (81.30 ± 14.15 vs. 76.55 ± 12.81°, *P* = 0.014) than the control group. Arteriolar tortuosity (*P* = 0.156) and arteriolar branching angles (*P* = 0.821) were not significantly different between the groups.

**Table 2:** Comparison of retinal vascular parameters between eyes with idiopathic epiretinal membrane and normal controls.

Retinal vascular parameter	Idiopathic ERM (98 eyes)	Controls (99 eyes)	*P* value*
Caliber			
CRAE (µm)	163.56 ± 14.15	158.61 ± 10.42	**0.006**
CRVE (µm)	211.64 ± 15.87	199.79 ± 15.71	**< 0.001**
Fractals			
Total fractal dimension	1.333 ± 0.059	1.355 ± 0.075	**0.022**
Arteriolar fractal dimension	1.146 ± 0.072	1.181 ± 0.092	**0.003**
Venular fractal dimension	1.107 ± 0.063	1.111 ± 0.072	0.638
Tortuosity			
Arteriolar tortuosity (× 10^–4^)	0.672 ± 0.167	0.641 ± 0.141	0.156
Venular tortuosity (× 10^–4^)	0.593 ± 0.137	0.544 ± 0.119	**0.008**
Bifurcation			
Arteriolar branching angle (°)	77.72 ± 17.32	77.18 ± 15.88	0.821
Venular branching angle (°)	81.30 ± 14.15	76.55 ± 12.81	**0.014**

*CRAE* central retinal arteriolar equivalent, *CRVE* central retinal venular equivalent, *ERM* epiretinal membrane.

Continuous variables are reported as mean ± standard deviation.

Significant factors appear in boldface.

*Student *t* test.

Table 3 shows the associations between idiopathic ERM and retinal vascular geometric measurements. Multivariate regression analysis using variables selected by backward stepwise logistic regression showed that female sex (odds ratio [OR] 0.402; 95% CI 0.196–0.802; *P* = 0.011), wider retinal venular caliber (OR 16.852; 95% CI 5.384–58.997; *P* < 0.001) and decreased total fractal dimension (OR 0.156; 95% CI 0.052–0.440; *P* = 0.001) were more likely to have idiopathic ERM.

**Table 3:** Associations between idiopathic epiretinal membrane and retinal vascular parameters.

Retinal vascular parameter	Univariate analysis			Multivariate analysis		
	OR	95% CI	*P* value	OR	95% CI	*P* value
Age	0.987	0.956, 1.017	0.396			
Sex	0.465	0.257, 0.831	**0.010**	0.402	0.196, 0.802	**0.011**
Hypertension	1.361	0.737, 2.533	0.326			
Diabetes mellitus	0.658	0.306, 1.383	0.274			
Hypertriglyceridemia	0.824	0.332, 2.008	0.670			
Hypercholesterolemia	1.711	0.876, 3.408	0.119			
Body mass index	0.997	0.895, 1.110	0.950			
Caliber						
CRAE, per 50 increase	5.112	1.61, 17.362	**0.007**	4.060	0.987, 17.881	0.057
CRVE, per 50 increase	10.866	4.151, 31.055	**0.000**	16.852	5.384, 58.997	**< 0.001**
Fractals						
Total fractal dimension, per 0.2 increase	0.373	0.155, 0.863	**0.024**	0.156	0.052, 0.440	**0.001**
Arteriolar fractal dimension, per 0.2 increase	0.359	0.175, 0.713	**0.004**			
Venular fractal dimension, per 0.2 increase	0.818	0.353, 1.880	0.636			
Tortuosity						
Arteriolar tortuosity, per 0.1 × 10^–3^ increase	3.842	0.614, 26.780	0.159			
Venular tortuosity, per 0.1 × 10^–3^ increase	21.745	2.245, 248.008	**0.010**	11.393	0.770, 200.722	0.085
Bifurcation						
Arteriolar branching angle, per 100 increase	1.217	0.223, 6.719	0.820			
Venular branching angle, per 100 increase	14.808	1.747, 144.553	**0.016**	9.738	0.780, 140.614	0.084

Significant values are in bold.

*CRAE* central retinal arteriolar equivalent, *CRVE* central retinal venular equivalent, *OR* odds ratio, *CI* confidence interval.

**Figure 1:**
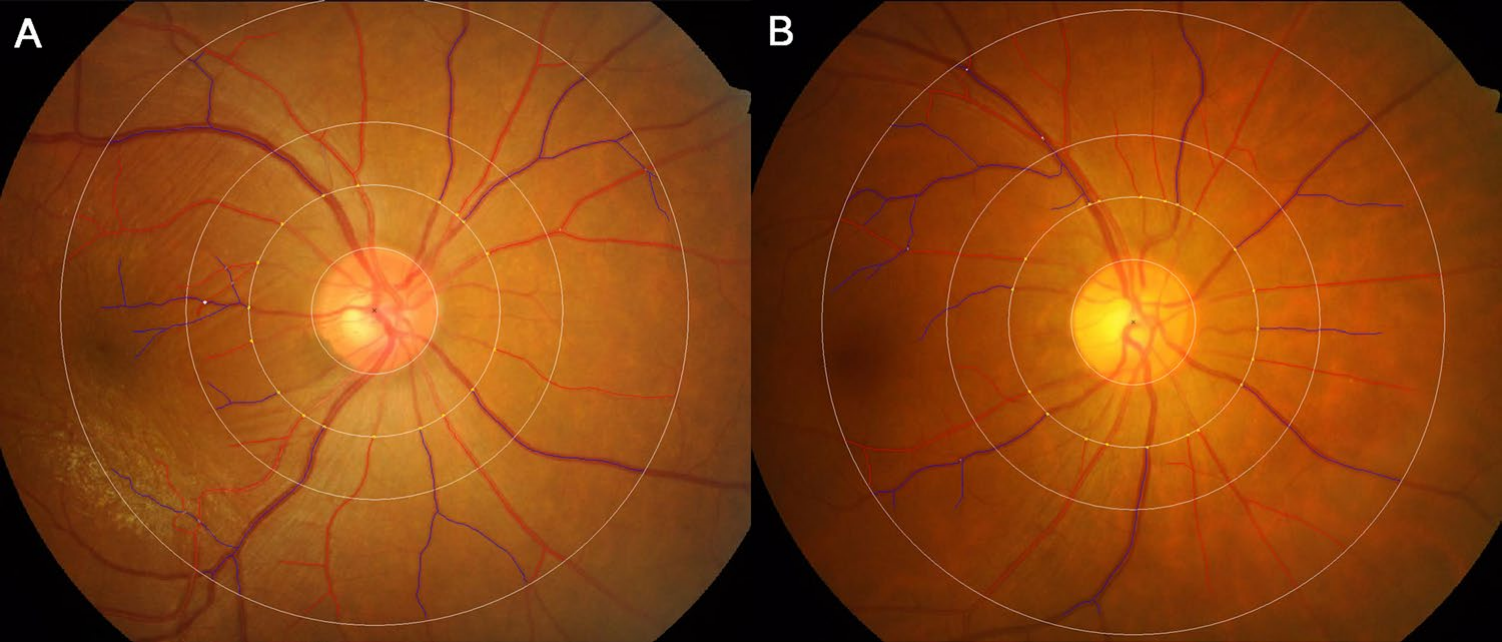
The geometric measurement of the retinal vasculature assessed by the Singapore I Vessel Assessment (SIVA) software of an eye with idiopathic epiretinal membrane (ERM) (**A**) and a normal control (**B**). (**A**) The retinal calibers of the arterioles and venules are 175.20 and 230.05 µm, respectively, the fractal dimensions of the arterioles and venules are 1.244 and 1.087, respectively, the tortuosity values of the arterioles and venules are 0.663 × 10^–4^ and 0.569 × 10^–4^, respectively, and the branching angles of the arterioles and venules are 65.14 and 71.86°, respectively. **B.** The retinal calibers of the arterioles and venules are 168.16 and 199.17 µm, respectively, the fractal dimensions of the arterioles and venules are 1.309 and 1.098, respectively, the tortuosity values of the arterioles and venules are 0.556 × 10^–4^ and 0.356 × 10^–4^, respectively, and the branching angles of the arterioles and venules are 62.79 and 76.76°, respectively.

Figure 1 demonstrates retinal microvasculature analyses performed using the Singapore I Vessel Assessment (SIVA) program, showing wider retinal vascular caliber, smaller retinal arteriolar fractal dimension, and higher retinal venular tortuosity in idiopathic ERM patients.

## Discussion

In the present study, we measured retinal vascular geometric parameters in eyes with idiopathic ERM and analyzed which retinal vessel geometric parameters are associated with idiopathic ERM. Our results demonstrate that compared with age-matched controls, and while adjusting for concomitant risk factors, eyes with idiopathic ERM are more likely to manifest structural changes in retinal microvascular network; wider retinal venules and smaller total fractal dimensions. To the best of our knowledge, our study is the first to comprehensively examine the direct association between idiopathic ERM and retinal vascular geometric parameters.

Previous epidemiological studies have reported inconsistent associations between idiopathic ERM and potential systemic cardiovascular risk factors. Miyazaki et al.^6^ demonstrated that serum cholesterol is significantly associated with ERM and Ng et al.^5^ showed that hypercholesterolemia and narrower arteriolar diameter are significantly associated with ERM. Furthermore, in the Singapore Malay Eye Study^7^, narrower retinal arteriolar diameter was found to be associated with ERM. However, in the Beaver Dam study, neither narrowing of the retinal arterioles nor a history of cardiovascular disease was associated with the presence of ERM^11^. In the present study, we observed that wider retinal venules are significantly associated with idiopathic ERM, independent of vascular risk factors. We assume that the wider retinal vascular caliber may be a result of disturbances in the hemodynamics of the retinal blood flow of eyes with idiopathic ERM. It has earlier been speculated that hypoxia may have a role in the formation of idiopathic ERMs^12–14^. Armstrong et al.^12^ have reported on the presence of vascular endothelial growth factor (VEGF) and tumor necrosis factor-α (TNF-α) not only in proliferative diabetic membranes, but in idiopathic ERMs as well. Lim et al.^14^ have described the presence of hypoxia-inducible factor-1α (HIF-1α), a transcription factor that plays an essential role in the systemic homeostasis response to hypoxia, in nondiabetic ERMs. The HIF-1 triggers the activation of several genes that result in the production of VEGF and other angiogenic factors^13^. It is possible that the presence of ERM—that is attributable to traction and/or shear stress applied to the retina—and associated hypoxic conditions affect retinal caliber. Moreover, we speculate that wider retinal venules may be related to the macular edema, which is often observed in eyes with idiopathic ERM. Nevertheless, the result of this study does not allow us to conclude that wider retinal venules are due to the ischemia and disturbances in the hemodynamics of retinal blood flow caused by traction in eyes with ERM.

Fractal dimension and branching angle reflect the status of the circulatory function of blood vessels. An optimal branching angle is associated with greater efficiency in blood flow with lower energy expenditure^15^. Given that the normal human retinal circulation is a “self-similar” and fractal pattern, fractal analysis may provide an objective, quantitative technique to evaluate retinal vessel geometry^16^. In previous studies, smaller retinal fractal dimension was found to be associated with systemic disease, including proliferative diabetic retinopathy^17^ and hypertension^9^. However, the relationship between fractal dimension and branching angle and idiopathic ERM has not been well studied. In the present study, smaller total and arteriolar fractal dimension and larger venular branching angle were significantly associated with idiopathic ERM. However, this significance was lost (except for total fractal dimension) after multivariate adjustment for confounding variables. A smaller fractal dimension represents less branching density, reflecting potential changes in blood flow or endothelial dysfunction in eyes with idiopathic ERM.

Tortuosity or curvature of the retinal vessels is also a crucial parameter that describes the geometric pattern of retinal vasculature, which may represent the state of the retinal microcirculation. Vascular tortuosity may be linked with tissue perfusion impairment as a complex response mechanism mediated by secretions from vascular endothelial cells. These vascular endothelial cells play an essential role in autoregulation of blood flow by producing vasoactive endothelial factors such as nitric oxide and endothelin^18^. These mediators stimulate angiogenesis and thus increase tortuosity, which subsequently promotes better tissue perfusion. In the present study, the finding that increased venular tortuosity is associated with idiopathic ERM was not significant after multivariate adjustment. Despite being insignificant, this trend may demonstrate a potential role for alterations in blood flow and changes in the geometric pattern of the retinal vasculature in the pathogenesis of idiopathic ERM.

There are recent studies that investigated foveal microvasculature using optical coherence tomography angiography (OCTA) in eyes with ERMs. Okawa et al.^19^ have reported that the foveal avascular zones of eyes with ERM were significantly smaller than those of the control eyes. Kim et al.^4^ have also described that compared with the fellow eyes, eyes with ERMs had a lower parafoveal vascular densities and smaller FAZ areas even after surgery. While OCTA allows visualization of retinal microvasculature, including capillaries, and can reveal vessel area density, vessel length density, and foveal avascular zone metrics, such as circularity index and size, it has limitations in quantitatively showing the geometry of retinal arterioles and venules, including retinal vascular caliber, fractal dimension, tortuosity, and bifurcation/branching angle. Moreover, the flow images of OCTA can be affected by overlying ERM and shown as dark shadow artifacts^20^. This interferes with reliable vascular analysis, in particular quantitative flow assessment as vessel density. Therefore, we believe that even in the era of OCTA, this study still has the advantage of confirming the vessel geometry with SIVA software. Nevertheless, it would be helpful to understand the pathogenesis of ERM and changes in vascular metrics if further studies could correlate functional changes in retinal blood vessels using OCTA, with vessel geometry by SIVA software.

Previous studies that involved the use of fluorescein angiography to image the macular regions of eyes with ERMs revealed a reduced mean capillary flow velocity in these eyes^21^. Kadonosono et al.^22,23^ have also evaluated the retinal capillary blood flow velocity in patients with ERM and reported that the mean capillary blood flow in the perifoveal area was reduced as well. Afterward, Shinoda et al.^24^ measured the tissue blood flow in the macular area using scanning laser Doppler flowmetry and reported that the mean blood flow was significantly lower in eyes with ERMs than in control eyes. Furthermore, they suggested that the pathological reduction of retinal capillary blood flow velocity reported by Kadonosono et al.^22,23^ may not be compensated for, even by the blood vessel dilation found in eyes with ERMs, to decrease the mean blood flow. These findings are consistent with our results, showing the changes that occur in the geometric pattern of the retinal vasculature of eyes with idiopathic ERM. It is assumed that there is a mutually exacerbating relationship between retinal vascular insufficiency and ERM. Coppe et al.^25,26^ reported a reduction in perifoveal blood flow in the unaffected fellow eye of unilateral ERM, suggesting that relative tissue hypoxia probably precedes ERM formation and contraction and stimulates activation of Müller cells, and thus suggests that retinal vascular insufficiency may contribute to ERM formation. In addition, in the pathogenesis of ERM, glial tissues are affected by various growth factors and cytokines as they gradually proliferate and contract, which are known to further disrupts macular structures and retinal vasculature^27^. It may be possible that Müller cells in the foveal center, which are stressed by the traction, release growth factors like fibroblast growth factor-2 (FGF2) and platelet-derived growth factor (PDGF) that stimulate astrocyte migration and proliferation and thus facilitate ERM formation^28,29^. Deformities in the retinal tissues and neurovascular components caused by ERM-induced tractional forces may further accelerate relative tissue hypoxia and lead to the retinal vascular geometry changes identified in the current study. Alterations in the retinal vasculature, wider retinal venules and smaller fractal dimensions, may further induce the hemodynamic disturbances in the microcirculation of eyes with ERM, even though the pathophysiologic mechanisms and a causality relationship between retinal vascular geometry and ERM remains unclear.

There are some limitations to our study. Firstly, the design of the study was retrospective and cross-sectional. Therefore, whether retinal vascular geometric changes are antecedent or consequent to idiopathic ERM cannot be determined from these data. Further longitudinal studies are needed to assess causality. Secondly, we only included eyes with idiopathic ERM, therefore, our results cannot be extended to eyes with secondary ERMs. The different causes and pathogenic mechanisms of idiopathic and secondary ERMs may result in not only differences in membrane characteristics but also in differences in retinal vascular geometry. Thirdly, differences in vascular geometry analysis based on the ERM stage were not included in this study. Govetto et al.^30^ categorized OCT-based ERM into stages 1 through 4. In the future, analyzing vascular parameters to reflect disease severity according to ERM stage may lead to a deeper understanding of ERM pathogenesis. Fourthly, although the structural pathology in patients with ERM predominantly involve the macula, the SIVA program analyzed retinal microvasculature taken centered on the optic disc. Lastly, fine vessels beneath the ERM with diameters smaller than 25 µm were not outlined, which may have affected the results of this study. In future study, further analysis with OCTA, which can analyze the retinal microvasculature, including the fine capillaries, may improve the clinical significance.

In conclusion, alterations in retinal vascular geometric parameters, particularly wider retinal venules and smaller fractal dimensions, were found to be associated with the presence of idiopathic ERM. This association was independent of cardiovascular risk factors. The results of our study suggest that there may be microvascular network changes in the retinas of patients with idiopathic ERM, which could potentially be related to hemodynamic disturbances in their microcirculation.

## Methods

### Study participants

This was a retrospective, cross-sectional study that included 98 eyes of 98 consecutive patients who presented at the Seoul National University Hospital with a diagnosis of idiopathic unilateral ERM from January 2015 to December 2018; 99 eyes of healthy age-matched individuals who were examined at the Seoul National University Hospital clinic were also included as controls. The study protocol was approved by the Institutional Review Board and the study conduct adhered to the tenets of the Declaration of Helsinki. Institutional Review Board of the Seoul National University Hospital waived the need for written informed consent from the participants, because of the study’s retrospective design.

ERM diagnosis was made by a physician via indirect fundus examination and confirmed with spectral-domain optical coherence tomography (SD-OCT). Subjects were excluded if any of the following were present: (1) secondary ERM associated with a history of uveitis, retinal vein occlusion, retinal detachment, proliferative diabetic retinopathy, retinal dystrophy, ocular trauma, or other underlying maculopathy; (2) high hyperopia (≥ + 5.0 diopters) or high myopia (≤ − 6.0 diopters); (3) macular edema due to retinal vascular diseases; (4) optical media opacity that could significantly interfere with image acquisition in fundus photography (e.g., cataract of more than grade III in the Emery–Little classification).

### Laboratory tests and examination

All participants underwent comprehensive ophthalmologic and systemic examinations, including collection of blood samples. Hemoglobin A1c (HbA1c), total serum cholesterol, triglyceride, low density lipoprotein cholesterol (LDL-cholesterol) and high-density lipoprotein cholesterol (HDL-cholesterol) levels were all measured. BMI was calculated as weight (in kilograms) divided by height (in meters) squared. Hypertension was defined as systolic blood pressure ≥ 140 mmHg, diastolic blood pressure ≥ 90 mmHg, or the current use of antihypertensive medication. Diabetes mellitus was defined as a random or post-load glucose level ≥ 11.1 mmol/l, use of antidiabetic medication, or self-reported history of diabetes. Hypertriglyceridemia was defined as triglyceride level ≥ 150 mg/dl (1.7 mmol/l) and hypercholesterolemia was defined as a total cholesterol level ≥ 220 mg/dl (5.69 mmol/l)^31^. Ophthalmologic examinations included slit-lamp biomicroscopy, indirect fundus examination, fundus photography (Vx-10; Kowa Optimed, Tokyo, Japan), and SD-OCT (Cirrus; Carl Zeiss Meditec, Dublin, CA).

### Retinal vessel geometry

Digital fundus photographs were obtained using a digital retinal camera (Vx-10; Kowa Optimed, Tokyo, Japan) in 45-degree mode centered on the optic disc. A semi-automated software (SIVA, cloud-based version, National University of Singapore, Singapore) was used to quantitatively measure the following retinal vascular geometric parameters from the digital photographs: retinal vascular caliber, retinal vascular fractal dimension, retinal vascular tortuosity, and retinal vascular branching pattern. Briefly, the program automatically detected and traced the optic disc and set the grading grid on the fundus photograph. It then outlined all retinal vessels (arterioles and venules) greater than 25 µm in diameter and generated a skeleton image of the retinal microvasculature. Two graders, masked to the identities and characteristics of the participants, assessed the fundus photographs for retinal vessel geometric measures. The graders were responsible for evaluation of the measurements acquired with SIVA according to a standardized grading protocol^32^. The measured area was the region between 0.5 and 2.0 disc diameters away from the disc margin.

Retinal vascular caliber was measured using the SIVA program, which followed the standardized protocol used in the Atherosclerosis Risk in Communities study^33^. The revised Knudtson–Parr–Hubbard formula was used to calculate average retinal arteriolar and venular calibers, which were presented as central retinal arteriolar equivalent (CRAE) and central retinal venular equivalent (CRVE), respectively^34^.

Total, arteriolar, and venular fractal dimensions were calculated from a skeletonized line tracing of the retinal vessels using the box-counting method, in which each photograph is divided into a series of squares of various side lengths^35^. These represent a “global” measure that summarizes the whole branching pattern of the retinal vascular tree^9^. A fractal can be defined as a geometric pattern that is “self-similar” and summarized by the fractal “dimension,” which measures the complexity of the branching pattern^9^. The fractal dimension is usually a ratio and has no units. Larger values indicate a more complex branching pattern.

Retinal vascular tortuosity was derived from the integral of the curvature square along the path of the vessel, normalized by the total path length^8^. The straighter the vessel, the lower the tortuosity value. The estimates were summarized as retinal arteriolar tortuosity and retinal venular tortuosity, representing the average tortuosity of the arterioles and venules of the eye, respectively.

Retinal vascular branching angle was defined as the first angle subtended between two daughter vessels at each vascular bifurcation^15^. Retinal arteriolar branching angle and retinal venular branching angle quantify the average branching angles of the arterioles and venules of the eye, respectively.

### Statistical analysis

Statistical analyses were performed using R software (version 2.13.0; Vienna, Austria). The Student’s *t* test and the chi-square test were used to compare the characteristics of the eyes with idiopathic ERM with those of the healthy controls. Factors related to the presence of idiopathic ERM were identified using univariate and multivariate analyses for those factors selected by the stepwise logistic regression.

## Data Availability

Data supporting the findings of the current study are available from the corresponding author on reasonable request.
